# Deep convolutional neural networks are sensitive to face configuration

**DOI:** 10.1167/jov.24.12.6

**Published:** 2024-11-05

**Authors:** Virginia E. Strehle, Natalie K. Bendiksen, Alice J. O’Toole

**Affiliations:** 1 School of Behavioral and Brain Sciences, The University of Texas at Dallas, Dallas, Texas, USA

**Keywords:** face perception, deep convolutional neural network, configuration, feature

## Abstract

Deep convolutional neural networks (DCNNs) are remarkably accurate models of human face recognition. However, less is known about whether these models generate face representations similar to those used by humans. Sensitivity to facial configuration has long been considered a marker of human perceptual expertise for faces. We tested whether DCNNs trained for face identification “perceive” alterations to facial features and their configuration. We also compared the extent to which representations changed as a function of the alteration type. Facial configuration was altered by changing the distance between the eyes or the distance between the nose and mouth. Facial features were altered by replacing the eyes or mouth with those of another face. Altered faces were processed by DCNNs (Ranjan et al., 2018; Szegedy et al., 2017) and the similarity of the generated representations was compared. Both DCNNs were sensitive to configural and feature changes—with changes to configuration altering the DCNN representations more than changes to face features. To determine whether the DCNNs' greater sensitivity to configuration was due to a priori differences in the images or characteristics of the DCNN processing, we compared the representation of features and configuration between the low-level, pixel-based representations and the DCNN-generated representations. Sensitivity to face configuration increased from the pixel-level image to the DCNN encoding, whereas the sensitivity to features did not change. The enhancement of configural information may be due to the utility of configuration for discriminating among similar faces combined with the within-category nature of face identification training.

## Introduction

To recognize and differentiate faces requires sensitivity to both facial features (e.g., eyes, nose) and their configural arrangement within a face (Tanaka & Gordon, 2011). The configural arrangement, referred to as second-order relational information, is defined by the spatial relationships between constituent face features (e.g., distance between the eyes) (Diamond & Carey, 1986). The ability to process and use configural information for recognition is thought to be a hallmark of perceptual expertise for faces, because it promotes “within-category discrimination” (Maurer, Le Grand, & Mondloch, 2002). Face recognition is a within-category task, because it entails discriminating individual identities from within the category of “faces.” Although the psychological literature on the perception of features and configuration in faces is vast, it converges on the idea that face configuration plays a critical role in human face perception (Diamond & Carey, 1986; Farah, Wilson, Drain, & Tanaka, 1998; Rossion, 2008; Tanaka & Farah, 1993; Young, Hellawell, & Hay, 1987; Yovel & Kanwisher, 2005). It is also clear from previous studies that features provide useful information for face recognition (e.g., Abudarham, Shkiller, & Yovel, 2019). From a broader perspective, the encoding of features depends on the configural context in which they appear, and the processing of features within a specified configuration is a prerequisite for creating a holistic representation of a face (Tanaka & Farah, 1993; Tanaka & Gordon, 2011; Tanaka & Sengco, 1997).

Despite general agreement on the importance of both features and face configuration for accurate face recognition, it is commonly argued that sensitivity to facial configuration underlies human face recognition at its best (e.g., Diamond & Carey, 1986). The ability to encode fine-scale differences in the relative placement of features in a face can be critical in differentiating highly similar faces. This skill is considered fundamental to human face processing expertise. Notably, these ideas about human facial expertise were developed in the literature decades ago when most studies used highly controlled images with little or no variability in image conditions (e.g., viewpoint, illumination) or appearance (e.g., expression, glasses). Traditional ideas about human expertise for faces have since evolved based on striking demonstrations of perceptual failures in perceiving face identity across variable images for unfamiliar faces (e.g., Jenkins, White, Van Montfort, & Burton, 2011). It is widely appreciated now that accurate face recognition requires both the ability to tell people apart (i.e., distinguish among highly similar faces) and the ability to “tell people together” (i.e., see identity constantly from multiple variable images) (Andrews, Jenkins, Cursiter, & Burton, 2015). The former refers to traditional ideas about facial expertise and the latter refers to the real-world challenge of managing image variability in the visual computation of a generalizeable representation of facial identity (e.g., Noyes et al., 2021).

In practical terms, computational models of face identification based on deep convolutional neural networks (DCNNs) have made enormous progress in solving these challenges. DCNNs now achieve high levels of accuracy on face identification tests (Ranjan, Sankaranarayanan, Castillo, & Chellappa, 2017; Taigman, Yang, Ranzato, & Wolf, 2014). Moreover, in human–machine comparisons, they perform at levels comparable with humans (Parde et al., 2023; Phillips et al., 2018; Taigman et al., 2014). They have proven effective both at telling similar faces apart (e.g., Parde et al., 2023) and at “perceiving” identity across variable images (e.g., Hill et al., 2019). For example, DCNNs can distinguish between the faces of identical twins and perform at a level comparable to the best human participants on the same task (Parde et al., 2023). Concomitantly, both human participants and a DCNN were capable of identifying twins over viewpoint-variable images. As expected, the performance of both humans and the DCNN decreased as the viewpoint offset between the two face images increased. In addition, there was reasonable accord between the similarity judgments made by humans and the deep network. Human judgments of whether two images were the same identity (rated on a 5-point Likert scale from sure the same person to sure different people) correlated with the DCNN's perceived similarity between the face images (measured as the cosine between the DCNN-generated representations). Significant human–machine correlations were found in six of the nine experimental conditions.

The ability of a DCNN to discriminate the faces of identical twins over variable images, and the rough parity in the similarity of the human and machine perception, raises the question of whether machines, like humans, represent facial configuration and/or facial features. There is evidence to indicate that face-trained DCNNs are sensitive to face features that play critical roles in human recognition of familiar faces (Abudarham et al., 2019). Moreover, DCNN-generated representations, though not constrained by features with semantic meanings (e.g., lip thickness), are nonetheless impacted by them. An analogous, but not identical, question has been addressed for DCNNs in the object recognition literature in the context of the ability of DCNNs to perceive global shape and texture information in objects. Specifically, object recognition DCNNs, trained with the ImageNet dataset (Russakovsky et al., 2015), have consistently shown limited sensitivity to the global shape of an object, and instead show a texture bias when classifying various object classes at a basic or subordinate level of classification (Baker, Lu, Erlikhman, & Kellman, 2018; Baker, Lu, Erlikhman, & Kellman, 2020; Geirhos et al., 2018). More concretely, it would appear that object recognition networks rely more on surface texture, such as the fur of a cat or the wrinkled skin of an elephant, than on the outline shape of the animal. This bias can be reversed when variations in texture are implemented into training. This forces the network to rely on alternative sources of information about the object (Geirhos et al., 2018). Nonetheless, the texture bias finding has been cited widely as an example of DCNN behavior at odds with human perception (Wichmann & Geirhos, 2023).

Facial configuration differs from global outline shape in fundamental ways, although they both rely on spatial relational information in the face or object. Given that face configuration, like global shape, draws on spatial relational information, it is worth asking whether face networks lack sensitivity to the spatial relational information in face configuration. Differences between global shape and face configuration are also worth noting. The global shape of an object has been operationally defined as its two-dimensional silhouette (Baker & Elder, 2022; Baker et al., 2018). Face configuration refers to the internal placement of features in a face. For objects, configuration might be akin to the relative positions of windows in a house, which is not particularly useful for the between-category task of classifying a house, as such. By contrast, facial configuration has been established as a critical part of the information humans perceive saliently in faces. That, along with the variation in features (eye color, shape; mouth shape, etc.), make up the perceptually salient information in faces.

The purpose of the current study was to investigate whether DCNNs are sensitive to the features and configuration of a face. We used a reverse engineering approach whereby we altered the features or configuration of a face and measured the extent to which the DCNN-generated representations differed depending on the type of alteration. For the facial feature alteration, we exchanged either the eyes or mouth between different faces. For the configuration, we altered either the relative distance between the eyes or the distance from the nose to the mouth. All face images were processed through a DCNN to generate a representation. Next, we compared the dissimilarity between the DCNN-generated representations of feature-altered face pairs and configurally altered face pairs.

Given the ability of DCNNs to recognize faces with high accuracy, and the similarity between human and DCNN judgments in challenging face identity-matching tests (e.g., Parde et al., 2023), we hypothesized that DCNNs should be sensitive to both the features of a face and the face configuration. We did not predict a strong difference in sensitivity to configuration versus feature information. This is because previous studies have reported differences in perceptual sensitivity for configuration versus features only when faces are presented upside down (Freire, Lee, & Symons, 2000; Le Grand, Mondloch, Maurer, & Brent, 2001; Tanaka & Sengco, 1997)—an effect known as the face inversion effect (Yin, 1969). Perceptual sensitivity to the alteration of features and face configuration should depend on both the relative magnitude of the two types of alterations in the original image (e.g., amount of face changed and extent to which it is changed) and to the effects of visual processing. Accordingly, to control for image-based effects, in a second simulation, we compared the effects of the configural and feature alterations in the pixel-based images (i.e., the input to the DCNN) and in the DCNN-generated representation. This comparison enabled us to determine how neural network processing alters image-based information about facial features and face configuration to produce a high-level visual representation of a face.

## Methods

### Stimuli

#### Images

The images showed frontal views of women (*n* = 144) selected from the Human ID Database (O’Toole et al., 2005). Face images were assigned to act as either a target face (*n* = 48) or a donor face (*n* = 96). The features and configuration of target faces were altered, and features from donor faces (i.e., eyes and mouth) were used to complete target face feature alterations. Two donor faces were assigned to each target face such that two versions of each feature alteration could be created.

#### Image alterations

**Figure 1.**
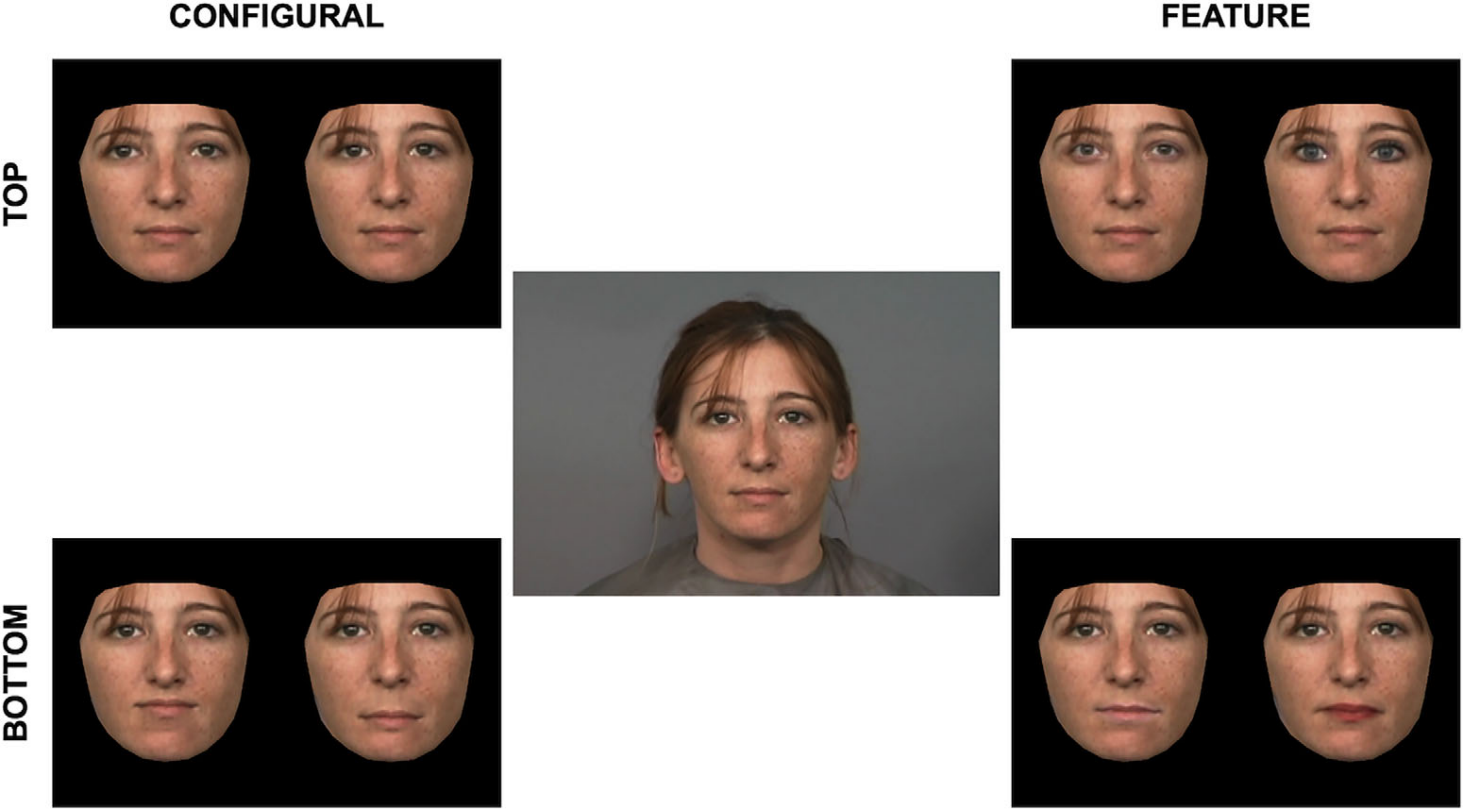
Configural alterations (left) changed the relative distances between a target face’s veridical features. Either the top of the face was altered by increasing or decreasing the space between the eyes (top left) or the bottom of the face was altered by translating the mouth up or down (bottom left). Feature alterations (right) exchanged a target face’s features with corresponding features from one of two associated donor faces. The top of the face was altered by exchanging the eyes (top right) or the bottom of the face was altered by exchanging the mouth (bottom right).

We used a popular method in the psychological literature used to illustrate the influence of features and configurations in human face processing to complete the feature and configural alterations (Freire et al., 2000; Le Grand et al., 2001; Tanaka & Sengco, 1997) (Figure 1).

Features and configurations positioned in the top and bottom regions of target faces were altered in-house using Adobe Photoshop (Adobe Inc., 2019). Configural alterations included 1) changing (i.e., increasing and decreasing) the distance between the eyes and 2) translating the mouth up and down. The distance between the eyes was altered using the liquify tool. The location of the mouth was altered by segmenting the mouth region with the lasso tool and moving it up or down on a pixel-by-pixel basis. Alterations to the configuration were done to the face until the experimenter deemed them to be just noticeably different from the original target image. However, there was no quantifiable limit to the degree of alteration. For the feature alterations, either the target's eyes or mouth were exchanged with one of two corresponding donor faces. Features were segmented using the lasso tool and placed over the target face's original feature. Techniques such as blending were used to minimize differences between the donor feature and the target face (e.g., stark differences in skin tone or lighting) that could reveal a perceptually noticeable discrepancy between the altered feature and the target face.

Eight versions of each target face were generated from both the configural and feature alterations, yielding a total of 384 altered images. Using the altered images, we created 192 opposite pairs to measure the salience of features and configurations in DCNN representations. Opposite pairs consisted of two images of the same target face with the same alteration type and location. The only difference between the images was the direction of the alteration (i.e., inward vs. outward eye spacing; upward vs. downward for the nose to mouth spacing). An example of an opposite pair would be two images of the same target face in which one image showed a mouth with thin neutral tone lips and the other features a mouth with full and bright-colored lips. Notice that the type of alteration (feature) and the location of the alteration (bottom) remains consistent between the two images, but the alteration directions (thin/neutral toned and full/bright colored) differ. Opposite pairs existed on all levels of alteration type and location for all target faces.

### Networks

We used two networks with markedly different architectures to be sure that the results were general rather than due to a specific type of DCNN. In previous work, the effects of alterations to the visual qualities of an image in DCNN representations typically replicate between different types of networks (cf., Hasson, Nastase, & Goldstein, 2020; O’Toole & Castillo, 2021). We used an Inception ResNet V1 model (Esler, 2020; Szegedy, Ioffe, Vanhoucke, & Alemi, 2017) pretrained with VGGFace2 and a ResNet network (Ranjan et al., 2018) pretrained with the Universe face dataset (Bansal, Castillo, Ranjan, & Chellappa, 2017) to collect DCNN-generated face representations. The Inception architecture is a wide network consisting of Inception modules. These modules consist of blocks of layers with varying convolution filter sizes (e.g., 1 × 1, 3 × 3). In contrast, the ResNet model is a deep network (27 convolutional layers, two fully connected layers) that uses a Crystal loss function.

### Experimental design

**Figure 2.**
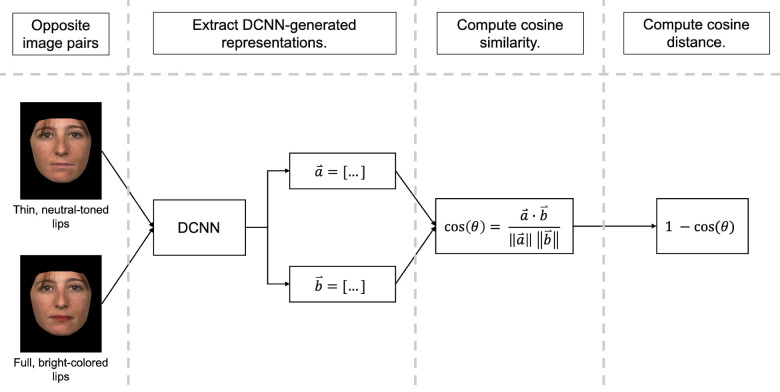
A schematic representation of the experimental design. We generated opposite image pair representations from the Inception and ResNet networks. Cosine between opposite pair representation vectors was computed. Dissimilarity of opposite pairs was measured using (1 − cosine). Image-based, pixel representations were generated by concatenating RGB values for each pixel within an image. Dissimilarity between image-based vectors was computed in the same way it was for DCNN representation vectors.

The study consisted of two simulations. In the first simulation, we assessed the salience of face features and their configuration in DCNN representations. Specifically, the DCNN representations of feature- and configurally altered face image pairs were extracted from the penultimate (fully connected) layers of the Inception and ResNet networks. Both representations were expressed as 512-element feature vectors. We compared the dissimilarity of opposite pair DCNN representations as a function of alteration type (feature, configural) and location (top, bottom) (Figure 2). Dissimilarity was computed using cosine distance (1 − cosine) between the representations of opposite pairs (cf., Dailey, Cottrell, Padgett, & Adolphs, 2002). With this measure, larger differences between opposite representations indicate greater impact with DCNN processing.

In the second simulation, we examined the salience of features and configurations in the raw input images fed to the DCNN by analyzing image-based, pixel representations. First, pixel representations of images from feature- and configurally altered opposite pairs were created using RGB values from each pixel in the altered images. We used the NumPy library in Python to extract RGB information from each pixel in the altered images. Next, we concatenated these values to create a single vector containing RGB information across the entire image. We used the method outlined elsewhere in this article to compute the dissimilarity of opposite pair pixel representations as a function of alteration type and location.

## Results

### Deep network representations of features and configurations

**Figure 3.**
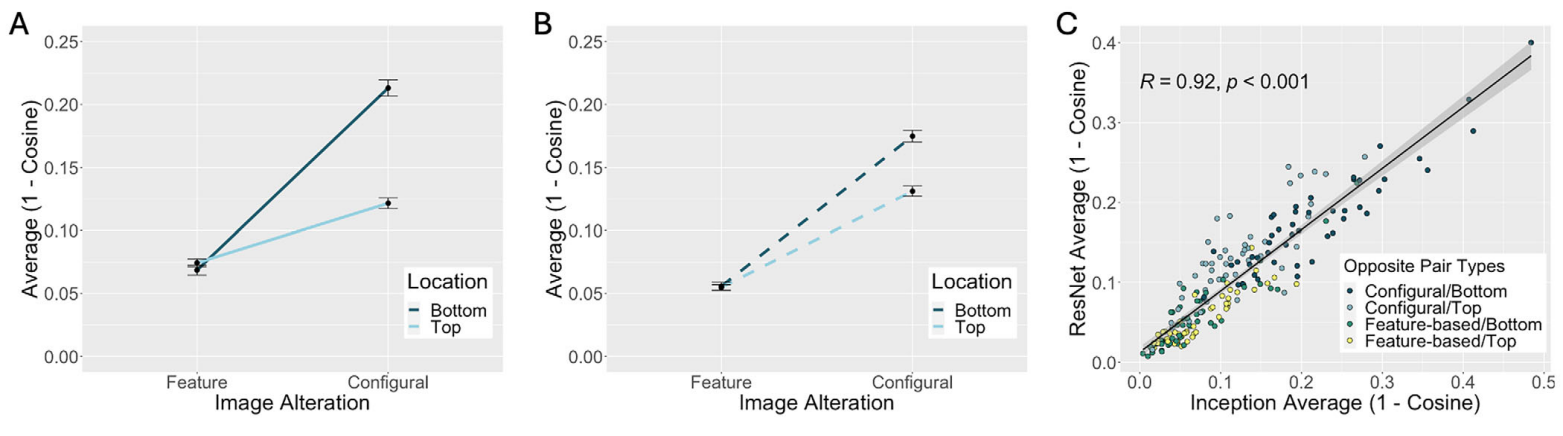
Opposite pair representations for both the Inception (A) and ResNet (B) networks were more dissimilar for configurally altered pairs, especially if the alteration was done to the bottom region of the face. Dissimilarity of opposite pair representations across the two networks were directly correlated despite differences in network architecture (C).

Figure 3 shows the dissimilarity (1 − cosine) between image pairs as a function of the type of alteration (feature versus configuration) and the location of the alteration (top vs. bottom of the face). Visual inspection of the pattern of results suggests four findings. First, all dissimilarities were substantially greater than than zero, indicating that both networks were sensitive to the feature and configural information in faces. Second, configural alterations yielded greater differences in DCNN representations than feature alterations, indicating that the DCNN perceived the configural changes more than the feature changes. Third, it is clear that alterations to the bottom of the face yielded larger differences than alterations to the top region of the face. Fourth, alteration type and location interacted. Configurally altered opposite pairs affected DCNN codes more than feature-altered pairs for alterations done to the bottom face.

More formally, the cosine data associated with each network were submitted to separate two-factor repeated-measures analyses of variance (ANOVAs) with alteration type (feature, configuration) and location (top, bottom) as the independent factors. The findings noted were supported statistically. First, there was a main effect of alteration type, such that configural alterations resulted in significantly higher dissimilarity between opposite pair representations in comparison to feature alterations, Inception: *F*(1, 47) = 164.73, *p* < 0.001, η^2^ = 0.78; ResNet: *F*(1, 47) = 206.05, *p* < 0.001, η^2^ = 0.81. Second, there was a main effect of alteration location, such that alterations to the bottom region of the face resulted in higher dissimilarity between opposite pair representations in comparison with alterations to the top region, Inception: *F*(1, 47) = 22.00, *p* < 0.001, η^2^ = 0.32; ResNet: *F*(1, 47) = 9.80, *p* = 0.003, η^2^ = 0.17. Third, there was a significant interaction between alteration type and location such that the effects of face location occurred only for configural alterations, Inception: *F*(1, 47) = 31.49, *p* < 0.001, η^2^ = 0.40; ResNet: *F*(1, 47) = 10.42, *p* = 0.002, η^2^ = 0.18.

At the level of individual stimuli, network perceptions of face dissimilarity between feature- and configurally altered opposite pairs correlated strongly, Pearson product moment correlation: *r*(190) = 0.92, *p* < 0.001, indicating accord in the effects of the alterations to the face features and configuration regardless of network architecture.

### Comparing low- and high-level representations

The first simulation showed that alterations to the facial configuration affected DCNN representations more than alterations to the face features. This might be due to characteristics of the DCNN encoding that emphasize information about face configuration over the features. Alternatively, it could be due to a priori differences in the images input to the DCNN. For example, the particular configural alterations we made to the faces may simply have changed the images more than the feature alterations we made. To test this, we examined the effects of feature and configural alterations in low-level pixel-based (i.e., RGB vectors) representations of opposite pairs and compared these representations to those produced by deep networks. This test also allowed us to examine more directly the effect that DCNN processing of the face images has on the representational salience of face configuration and features. Recall that the pixel-based images are the input to the DCNN. Therefore, in comparing the pixel and DCNN representations, we can gain direct insight into the effects of deep network processing of the features and facial configuration.

**Figure 4.**
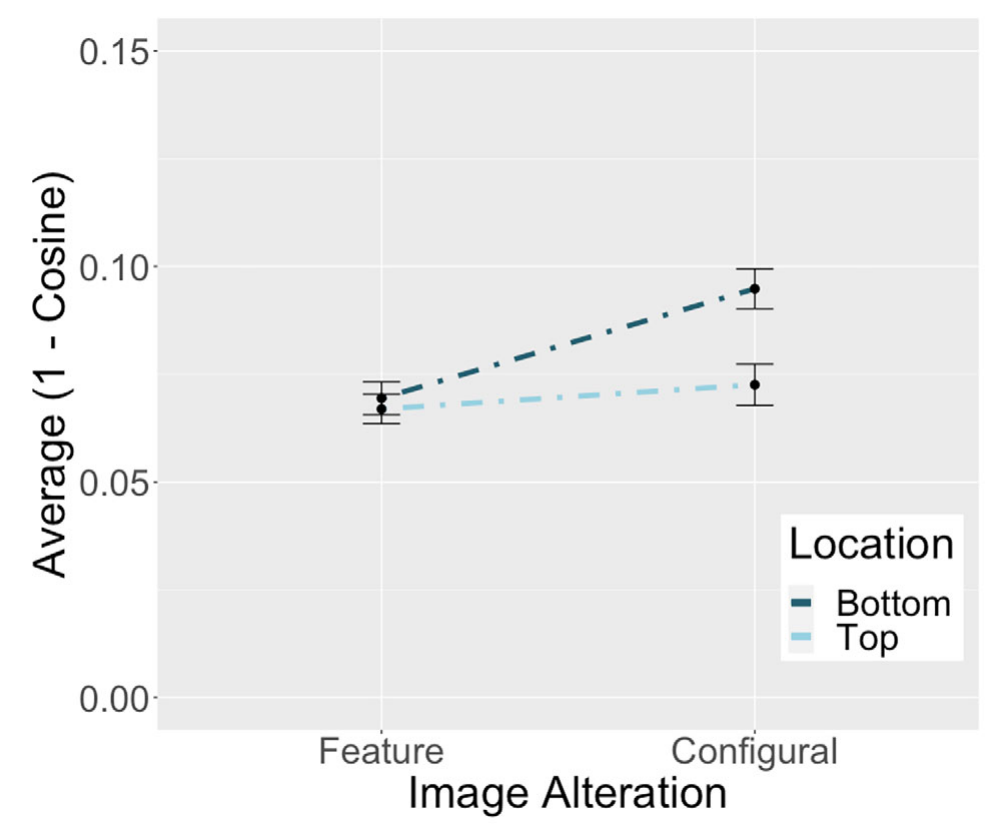
Average dissimilarity between pixel representations of opposite pairs. Configural alterations yield larger pixel differences than feature alterations. These differences exist prior to deep network processing.

Figure 4 illustrates the pattern of dissimilarities for the pixel representations as a function of alteration type and location. At the level of the image, the subsequent RGB vectors of opposite pairs with configural alterations differed more than those with feature alterations—at least for changes to the bottom of the face (although the latter is not supported statistically, given the lack of interaction; as discussed elsewhere in this article). The cosine data associated with the pixel representations were submitted to a two-factor repeated-measures ANOVA with alteration type (feature, configuration) and location (top, bottom) as independent factors. There was a significant main effect of alteration type, indicating that pixel representations differed more for configurally altered opposite pairs than for feature-altered pairs before *F*(1, 47) = 5.52, *p* = 0.023, η^2^ = 0.105. There was no effect of location, and no interaction between location and alteration type.

**Figure 5.**
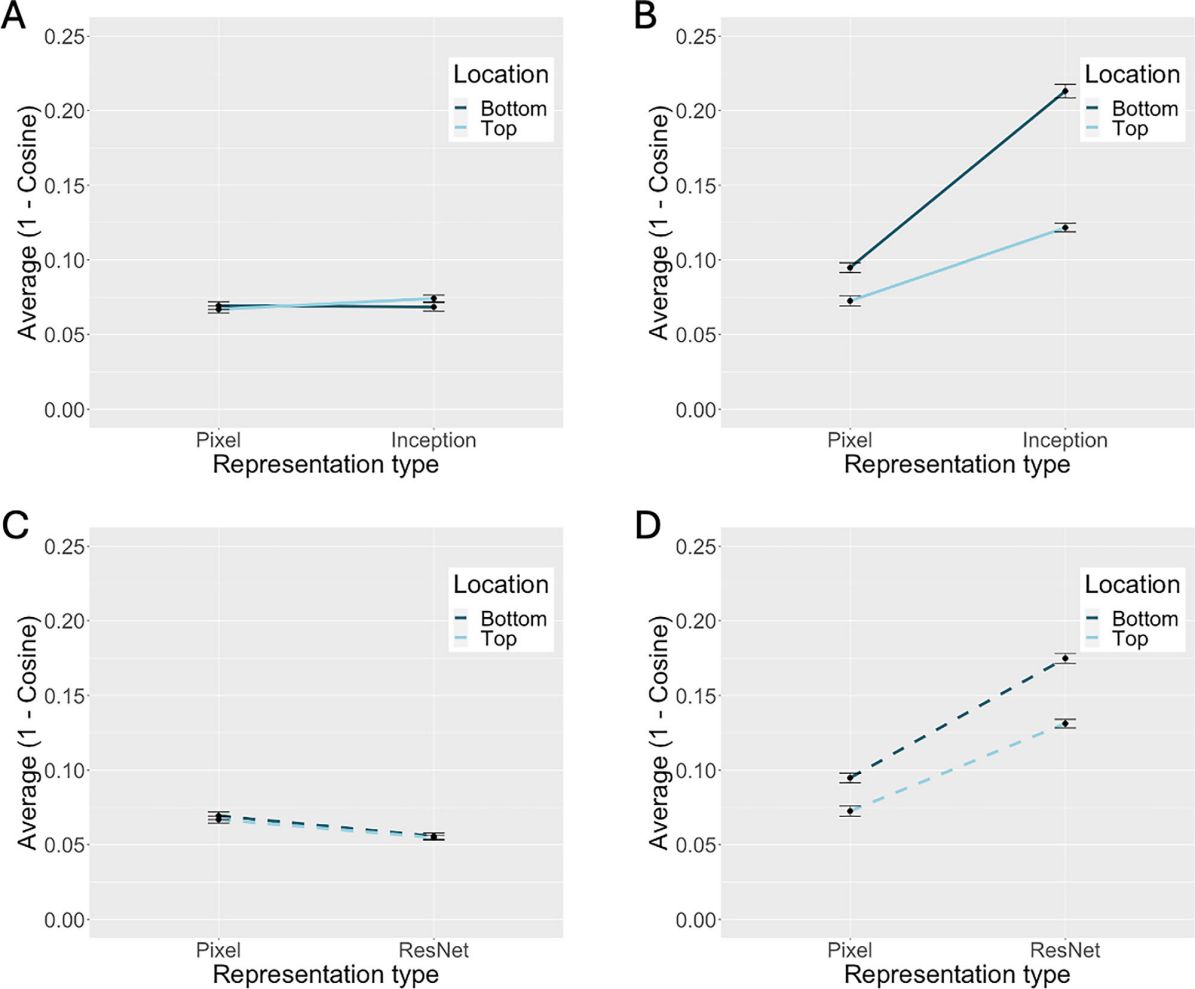
Alterations to facial features and configuration in pixel and DCNN representations. For feature alterations (A and C), distance between opposite pairs is comparable in pixel and DCNN representations. For configural alterations (B and D), DCNN representations of opposite pairs are more dissimilar than pixel representations.

These results indicate that there were a priori differences in the images input to the network, such that the configural alterations changed the images more than the feature alterations. However, in comparing the pattern of results for the pixel and DCNN representations, it is clear that these a priori image differences cannot account for the DCNN results. Figure 5 shows the pattern of dissimilarities for the feature and configural alterations in both the pixel and DCNN representations. It is clear from Figure 5 that DCNN processing did not affect the dissimilarity of opposite pairs with feature alterations. By contrast, DCNN processing strongly affected the dissimilarity of opposite pairs with configural alterations. Therefore, DCNN processing enhances the importance of configuration relative to the features, by making the opposite configuration pairs more dissimilar (distinct).

To test these processing effects more formally, the dissimilarities from the pixel and DCNN representations of opposite pairs were submitted to separate three-factor mixed ANOVAs (one for each network) with alteration type (feature, configuration), location (top, bottom), and representation type (pixel, DCNN) as the independent factors. For simplicity, we refer to the analyses as the Inception (Figures 5A and 5B) and ResNet analyses (Figures 5C and 5D), although both include comparisons between a network's representations and the pixel representations. Note that the pixel representations are the same in both analyses. We begin by presenting the effects that replicate across the inception and ResNet analyses.

A significant main effect of representation type revealed that the DCNN representations of opposite pairs were more different than the pixel representations, Inception: *F*(1, 47) = 46.10, *p* < 0.001, η^2^ = 0.50; ResNet: *F*(1, 47) = 24.89, *p* < 0.001, η^2^ = 0.35. Although this result suggests that DCNN processing decreased the similarity of opposite pair representations, a higher order interaction between alteration type and representation type qualifies this main effect to indicate that the decrease in similarity holds only for the configural alteration. Specifically, there was a significant interaction between alteration type and representation type for both networks, Inception: *F*(1, 47) = 79.15, *p* < 0.001, η^2^ = 0.63; ResNet: *F*(1, 47) = 103.73, *p* < 0.001, η^2^ = 0.69.

Consistent with the first simulation, there was a main effect of alteration type indicating that representations of configurally altered pairs differed more than the representations of feature-altered pairs, Inception: *F*(1, 47) = 105.90, *p* < 0.001, η^2^ = 0.69; ResNet: *F*(1, 47) = 111.70, *p* < 0.001, η^2^ = 0.70. There was also a main effect of alteration location, with higher dissimilarities for alterations to the bottom of the face, Inception: *F*(1, 47) = 17.94, *p* < 0.001, η^2^ = 0.28; ResNet: *F*(1, 47) = 9.19, *p* = 0.004, η^2^ = 0.16. Finally, there was a significant interaction between alteration type and location. This highlighted the higher impact of configural alterations to the bottom versus top of the face, Inception: *F*(1, 47) = 18.93, *p* < 0.001, η^2^ = 0.29; ResNet: *F*(1, 47) = 7.05, *p* = 0.011, η^2^ = 0.13. Location did not affect the dissimilarity of the feature representations.

**Figure 6.**
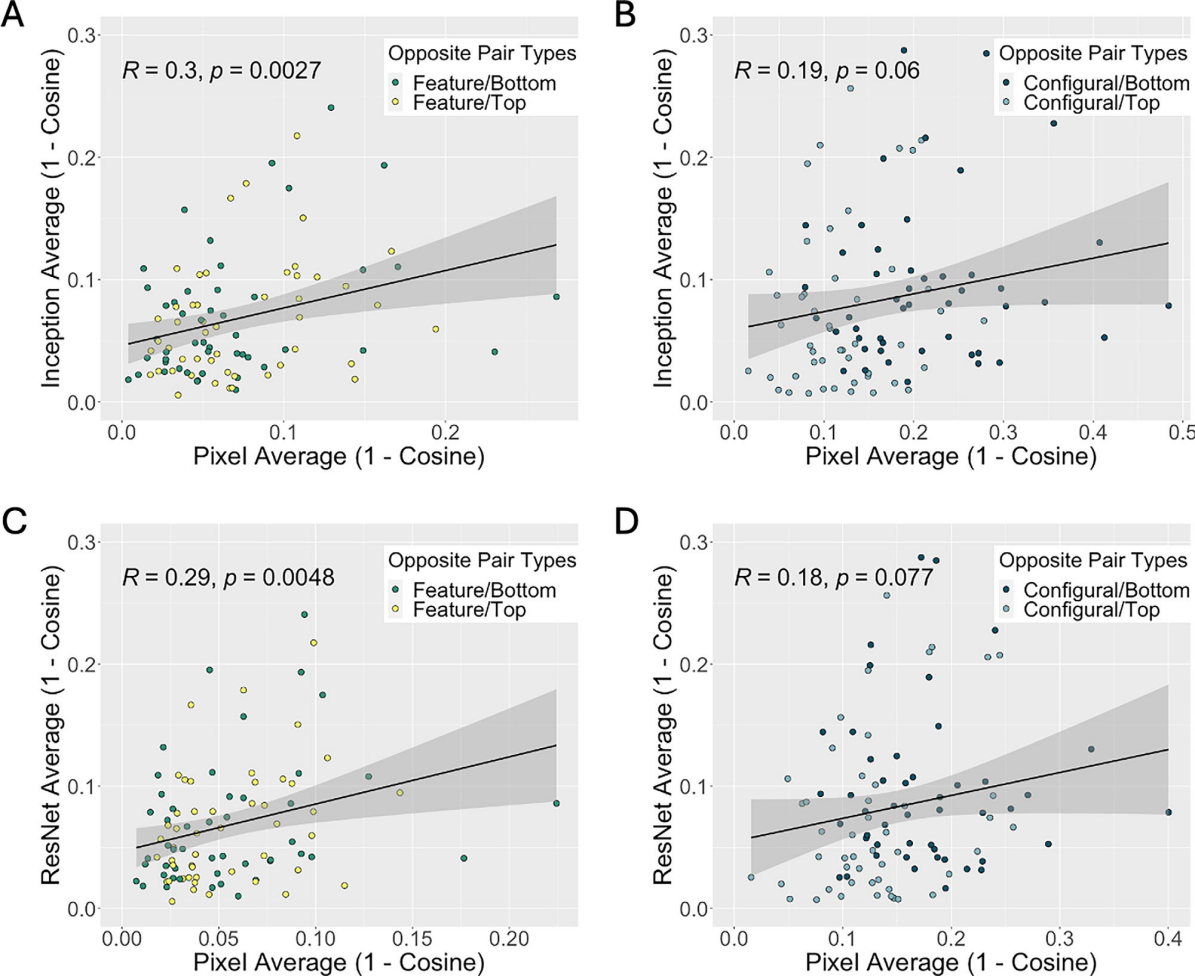
Scatter plots represent the correlation of opposite pair distances between pixel and DCNN representations (top = Inception, bottom = ResNet). The correlations are low for both the feature (A and C) and configural (B and D) alterations. This indicates that DCNN processing goes beyond a priori differences present at the pixel level.

Next, we examined the relationship between opposite pair distances on an item-by-item level to gain deeper insight into DCNN processing of feature and configural information. Specifically, we computed the Pearson product moment correlation between opposite pair pixel and DCNN representation distances as a function of alteration type (feature, configural). Figure 6 shows scatter plots that illustrate the correlation between opposite pair pixel and DCNN representation distances for the Inception (A and B) and ResNet (C and D) networks. Overall, the correlations are low for both feature, Inception: *r*(94) = 0.30, *R*^2^(94) = 0.09, *p* = 0.002679; ResNet: *r*(94) = 0.29, *R*^2^(94) = 0.0841, *p* = 0.004825, and configural alterations, Inception: *r*(94) = 0.19, *R*^2^(94) = 0.0361, *p* = 0.0603; ResNet: *r*(94) = 0.18, *R*^2^(94) = 0.0324, *p* = 0.0772, which corroborates our finding that DCNN processing goes beyond a priori differences present at the pixel level. Moreover, the correlations trend larger for feature alterations compared to configural alterations, although this is not supported statistically (Fisher’s r-to-z transformation) for either the Inception, *z* = 0.8, *p* = 0.2119, or ResNet, *z* = 0.79, *p* = 0.2148 networks.

For completeness, we report, but do not interpret, two additional interactions that occurred only in the Inception analysis. First, alteration location and representation type showed a significant interaction, Inception: *F*(1, 47) = 6.23, *p* = 0.016, η^2^ = 0.12. Second, there was a three-way interaction between alteration type, location, and representation type, Inception: *F*(1, 47) = 14.24, *p* < 0.001, η^2^ = 0.23.

In summary, despite a priori differences in the magnitude of configuration versus feature alteration in the images input to the network, these differences did not account completely for the DCNN representations' high sensitivity to configural information. Instead, as illustrated in Figure 5, sensitivity to face configuration is enhanced as a result of DCNN processing, whereas sensitivity to the features is unchanged.

## General discussion

There is long-standing evidence in the face perception literature that sensitivity to facial configuration plays an important role in people's ability to differentiate among similar faces. Although the role of features in face perception has received less attention, it is clear that facial features are also a critical part of human face representations (Abudarham et al., 2019). Computational models of the human face processing system should likewise demonstrate sensitivity to both types of information. In the present study, we found that two face-trained DCNNs (Inception and ResNet) with quite different architectures, detected alterations of facial configuration and face features. We conclude, therefore, that DCNNs, like humans, represent local features and global face configuration (Freire et al., 2000; Le Grand et al., 2001; Tanaka, Kay, Grinnell, Stansfield, & Szechter, 1998).

Considering this finding more closely, it is perhaps not surprising that face-trained DCNNs are sensitive to facial features. Previous work has demonstrated the predominance of local texture information in representations generated by object classification DCNNs (Geirhos et al., 2018). Sensitivity to facial configuration, however, is perhaps less expected, given the limited sensitivity of object classification networks to global shape (Geirhos et al., 2018). Facial configuration, like global shape, references the spatial layout of the image, thereby requiring a network or visual system to encode the global structure of an image. For face recognition networks, the present study demonstrates that information about the spatial layout of features, not only survives DCNN processing, but is enhanced by it.

One explanation for differences in the sensitivity of object versus face recognition networks to the global structural information in an image is that face and object recognition are fundamentally different tasks. Critically, object recognition is a between-category discrimination task, whereas face recognition is a within-category discrimination task. This distinction is well-understood in the psychological and visual neuroscience literature (e.g., Gauthier, Tarr, Anderson, Skudlarski, & Gore, 1999; Kanwisher, 2000), as well as in the computational literature (Dobs, Yuan, Martinez, & Kanwisher, 2023; Tong, Joyce, & Cottrell, 2008). The training of object recognition networks aims to map different exemplars of each category of object (e.g., chair, table) onto a single category-specific response. The training of face recognition networks aims to map different images of individual identities onto a single identity-specific response.

To better understand the relationship between face and object recognition in a deep network, it is worth noting that nearly all facial recognition networks are first trained on the task of object recognition, before being “retrained” or “tuned” (with transfer learning) for the task of face identification (O’Toole & Castillo, 2021). This secondary training adapts an object recognition network to the within-category task of discriminating individual identities. Learning individual identities enables the network to tell large numbers of similar people apart. This is the component of the face recognition process thought to be supported strongly by configural processing (Maurer et al., 2002). By contrast, for object recognition networks, separating individual exemplars of an object into different categories is an error. A common denominator of the tasks solved by face and object recognition networks is the problem of generalized categorization (face identity, object category) from variable images (view, illumination, etc.) of the same item (face or object). For faces, this is the “telling faces together” part of the face recognition task.

In turning to the relative importance of face features versus configuration, we found that alterations to face configuration changed DCNN representations more than alterations to face features. In isolation from the image-based information, this finding cannot be interpreted, because the DCNN-generated representation reflects both the input image and the DCNN processing. The image-based pixel analysis showed that at least part of the difference in the present case came from a priori differences in images. We made larger changes to face images in the configuration condition than in the feature condition. Although it would have been possible to control the number of pixels in each alteration type, this would not have produced natural-looking faces in all cases. Even so, direct evidence for the importance of configuration in DCNN descriptors comes instead from comparing the image-based and DCNN representations. Here we found that representational differences for configural alterations were enhanced by DCNN processing and remained unchanged for feature alterations.

One intriguing question concerns where in the network configural information was enhanced. In a previous study (Dobs, Yuan, Martinez, & Kanwisher, 2022), visualization of layer response profiles suggested that midlevel processing stages in a DCNN seem to process face features, whereas later stages seem to process faces more holistically. This might have relevance for modeling results from neurophysiological recordings and/or functional neuroimaging data. In practical terms, however, individual network layers in the present study would be difficult to interpret, because we tested two networks with fundamentally different architectures. Both networks enhanced configural information, without altering feature information. Consequently, interpretations about the significance of where in the network these differences emerge would be tentative. Notably, computational neuroscience theory (Hasson, Nastase, & Goldstein, 2020) and empirical findings (O’Toole & Castillo (2021)) point to the replicability of results in basic vision across a variety of network architectures, given networks with adequate computational capacity.

In summary, we found the DCNNs trained for face identification were sensitive to face features and their configuration. Network processing enhanced the representation of configuration, whereas the representation of face features was unaffected. The results are consistent with the perceptual importance face configuration plays in human face recognition. Future studies are needed to directly compare human and DCNN sensitivity to face configuration to understand the degree of similarity between the two systems.
